# Supplementary material for: COZOID: contact zone identifier for visual analysis of protein-protein interactions
Source: BMC Bioinformatics. 2018 Apr 6;19:125. doi: 10.1186/s12859-018-2113-6 (PMC5889581; doi:10.1186/s12859-018-2113-6)
Supplement: Supplementary file 2 — Software build. Executable binary file of the software tool. (ZIP 118,784 kb) [file 12859_2018_2113_MOESM2_ESM.zip › cozoid/java/docs/beansbinding-1.2.1-doc.zip!/org/jdesktop/observablecollections/ObservableMap.html]

ObservableMap (Beans Binding)


---


|  |  |  |
| --- | --- | --- |
| **Overview** \| **Package** \| **Class** \| **Use** \| **Tree** \| **Deprecated** \| **Index** \| **Help** | |  |
| **PREV CLASS**   **NEXT CLASS** | **FRAMES**    **NO FRAMES**     **All Classes** |  |
| SUMMARY: NESTED \| FIELD \| CONSTR \| METHOD | DETAIL: FIELD \| CONSTR \| METHOD |  |


---


## org.jdesktop.observablecollections Interface ObservableMap<K,V>

**All Superinterfaces:**: java.util.Map<K,V>

---

``` public interface ObservableMap<K,V> extends java.util.Map<K,V> ```

A `Map` that notifies listeners of changes to the `Map`.

---

**Nested Class Summary**

**Nested classes/interfaces inherited from interface java.util.Map**

`java.util.Map.Entry<K,V>`


| **Method Summary** | |
| --- | --- |
| `void` | `addObservableMapListener(ObservableMapListener listener)`             Adds a listener to this observable map. |
| `void` | `removeObservableMapListener(ObservableMapListener listener)`             Removes a listener from this observable map. |

**Methods inherited from interface java.util.Map**

`clear, containsKey, containsValue, entrySet, equals, get, hashCode, isEmpty, keySet, put, putAll, remove, size, values`

**Method Detail**

### addObservableMapListener

```
void addObservableMapListener(ObservableMapListener listener)
```

:   Adds a listener to this observable map.

    :   **Parameters:**: `listener` - the listener to add

---


### removeObservableMapListener

```
void removeObservableMapListener(ObservableMapListener listener)
```

:   Removes a listener from this observable map.

    :   **Parameters:**: `listener` - the listener to remove


---


|  |  |  |
| --- | --- | --- |
| **Overview** \| **Package** \| **Class** \| **Use** \| **Tree** \| **Deprecated** \| **Index** \| **Help** | |  |
| **PREV CLASS**   **NEXT CLASS** | **FRAMES**    **NO FRAMES**     **All Classes** |  |
| SUMMARY: NESTED \| FIELD \| CONSTR \| METHOD | DETAIL: FIELD \| CONSTR \| METHOD |  |


---
